# Deep brain stimulation for refractory obsessive-compulsive disorder (OCD): emerging or established therapy?

**DOI:** 10.1038/s41380-020-00933-x

**Published:** 2020-11-03

**Authors:** Hemmings Wu, Marwan Hariz, Veerle Visser-Vandewalle, Ludvic Zrinzo, Volker A. Coenen, Sameer A. Sheth, Chris Bervoets, Matilda Naesström, Patric Blomstedt, Terry Coyne, Clement Hamani, Konstantin Slavin, Joachim K. Krauss, Kai G. Kahl, Takaomi Taira, Chencheng Zhang, Bomin Sun, Hiroki Toda, Thomas Schlaepfer, Jin Woo Chang, Jean Régis, Rick Schuurman, Michael Schulder, Paresh Doshi, Philip Mosley, Anujan Poologaindran, Gabriel Lázaro-Muñoz, Joshua Pepper, Gaston Schechtmann, Anders Fytagoridis, Daniel Huys, Antonio Gonçalves-Ferreira, Pierre-François D’Haese, Joseph Neimat, Giovanni Broggi, Osvaldo Vilela-Filho, Jürgen Voges, Ahmed Alkhani, Takeshi Nakajima, Raphaelle Richieri, Diana Djurfeldt, Philippe Fontaine, Roberto Martinez-Alvarez, Yasushi Okamura, Jennifer Chandler, Katsushige Watanabe, Juan A. Barcia, Blanca Reneses, Andres Lozano, Loes Gabriëls, Antonio De Salles, Casey H. Halpern, Keith Matthews, Joseph J. Fins, Bart Nuttin

**Affiliations:** 1 Department of Neurosurgery, Second Affiliated Hospital, Zhejiang University School of Medicine, Hangzhou, Zhejiang, China; 2 Department of Clinical Neuroscience, Umeå University, Umeå, Sweden; 3 Department of Stereotactic and Functional Neurosurgery, University of Cologne, Cologne, Germany; 4 Department of Clinical & Motor Neurosciences, UCL Queen Square Institute of Neurology, 33 Queen Square, London, WC1N 3BG UK; 5 Department of Stereotactic and Functional Neurosurgery, Freiburg University Medical Center, Freiburg, Germany; 6 Department of Neurological Surgery, Baylor College of Medicine, Houston, TX USA; 7 Adult Psychiatry, Obsessive Compulsive Disorders Unit, UPC, and Department of Neuroscience, KU Leuven, Leuven, Belgium; 8 Department of Clinical Sciences/Psychiatry, Umeå University, Umeå, Sweden; 9 Unit for Deep Brain Stimulation, Umeå University, Umeå, Sweden; 10 Queensland Brain Institute, University of Queensland, Brisbane, Australia; 11 Division of Neurosurgery, Sunnybrook Health Sciences Centre, University of Toronto, Toronto, ON Canada; 12 Department of Neurosurgery, University of Illinois, Chicago, IL USA; 13 Department of Neurosurgery, Medical School Hannover, MHH, Hannover, Germany; 14 Department of Psychiatry, Social Psychiatry, and Psychotherapy, Medical School Hannover, MHH, Hannover, Germany; 15 Department of Neurosurgery, Tokyo Women’s Medical University, Tokyo, Japan; 16 Department of Functional Neurosurgery, Ruijin Hospital, Shanghai Jiao Tong University School of Medicine, Shanghai, China; 17 Department of Neurosurgery, Tazuke Kofukai Medical Research Institute and Kitano Hospital, Osaka, Japan; 18 Department of Interventional Biological Psychiatry, Freiburg University Medical Center, Freiburg, Germany; 19 Department of Neurosurgery and Brain Research Institute, Yonsei University College of Medicine, Seoul, South Korea; 20 Aix Marseille University, Inserm UMR1106, Department of Functional and Stereotactic Neurosurgery, Public Assistance Marseille Hospitals, Marseille, France; 21 Department of Neurosurgery, Academic Medical Center, Amsterdam, The Netherlands; 22 Zucker School of Medicine at Hofstra/Northwell, New York, NY USA; 23 Jaslok Hospital and Research Centre, Mumbai, India; 24 Systems Neuroscience Laboratory, QIMR Berghofer Medical Research Institute, Australia and the Queensland Brain Institute, St Lucia, QLD Australia; 25 Brain Mapping Unit, Department of Psychiatry, University of Cambridge, Cambridge, UK; 26 The Alan Turing Institute, British Library, London, UK; 27 Center for Medical Ethics and Health Policy, Baylor College of Medicine, Houston, TX 77030 USA; 28 Department of Neurosurgery, Queen Elizabeth Hospital, Birmingham, UK; 29 Department of Neurosurgery, Karolinska Institutet and University Hospital, Stockholm, Sweden; 30 Department of Psychiatry and Psychotherapy, University Hospital of Cologne, Cologne, Germany; 31 University Clinic of Neurosurgery, Faculdade Medicina Lisboa/Hospital Santa Maria, Lisbon, Portugal; 32 Vanderbilt School of Engineering, Nashville, TN USA; 33 University of Louisville, Louisville, KY USA; 34 Fond Istituto Neurologico C Besta, Milano, Italy; 35 Stereotactic and Functional Neurosurgery Service, Department of Neurosurgery, Medical School, Clinics Hospital, Federal University of Goiás, Goiânia, Brazil; 36 Department of Stereotactic Neurosurgery, Otto-von-Guericke University Magdeburg and Leibniz-Institute for Neurobiology, Magdeburg, Germany; 37 Division of Neurosurgery, King Abdulaziz Medical City, Riyadh, Saudi Arabia; 38 Department of Neurosurgery, Jichi Medical University, Tochigi, Japan; 39 Department of Psychiatry, Public Assistance Marseille Hospitals, Marseille, France; 40 Department of Clinical Neuroscience, Karolinska Institutet, Stockholm, Sweden; 41 Department of Psychiatry, CHC (Centre Hospitalier Chrétien), Liège, Belgium; 42 Unidad de Radiocirugía del Hospital Ruber Internacional de Madrid, Madrid, Spain; 43 Department of Neurosurgery, Tokyo Metropolitan Matsuzawa Hospital, Tokyo, Japan; 44 University of Ottawa Centre for Health Law, Policy and Ethics, Ottawa, ON Canada; 45 Department of Neurosurgery, Tokyo Metropolitan Matsuzawa Hospital, Kamikitazawa, Setagaya, Tokyo, Japan; 46 Department of Neurosurgery, Universidad Complutense de Madrid, Madrid, Spain; 47 Institute of Psychiatry and Mental Health, Complutense University de Madrid, Madrid, Spain; 48 Department of Neurosurgery, University of Toronto, Toronto, ON Canada; 49 Leo Schreursvest 13, 3001 Heverlee, Belgium; 50 Department of Neurosurgery, University of California, Los Angeles, David Geffen School of Medicine, Los Angeles, CA USA; 51 NeuroSapiens, Sao Paulo, Brazil; 52 Department of Neurosurgery, Stanford University Hospital, Stanford, CA USA; 53 Division of Molecular and Clinical Medicine, School of Medicine, University of Dundee, Ninewells Hospital & Medical School, Dundee, Scotland UK; 54 Division of Medical Ethics, Weill Cornell Medical College and New York Presbyterian Weill Cornell Medical Center, New York, USA; 55 Department of Neurosurgery, University Hospitals Leuven, KU Leuven, Leuven, Belgium

**Keywords:** Psychiatric disorders, Depression

## Abstract

A consensus has yet to emerge whether deep brain stimulation (DBS) for treatment-refractory obsessive-compulsive disorder (OCD) can be considered an established therapy. In 2014, the World Society for Stereotactic and Functional Neurosurgery (WSSFN) published consensus guidelines stating that a therapy becomes established when “at least two blinded randomized controlled clinical trials from two different groups of researchers are published, both reporting an acceptable risk-benefit ratio, at least comparable with other existing therapies. The clinical trials should be on the same brain area for the same psychiatric indication.” The authors have now compiled the available evidence to make a clear statement on whether DBS for OCD is established therapy. Two blinded randomized controlled trials have been published, one with level I evidence (Yale-Brown Obsessive Compulsive Scale (Y-BOCS) score improved 37% during stimulation on), the other with level II evidence (25% improvement). A clinical cohort study (*N* = 70) showed 40% Y-BOCS score improvement during DBS, and a prospective international multi-center study 42% improvement (*N* = 30). The WSSFN states that electrical stimulation for otherwise treatment refractory OCD using a multipolar electrode implanted in the ventral anterior capsule region (including bed nucleus of stria terminalis and nucleus accumbens) remains investigational. It represents an emerging, but not yet established therapy. A multidisciplinary team involving psychiatrists and neurosurgeons is a prerequisite for such therapy, and the future of surgical treatment of psychiatric patients remains in the realm of the psychiatrist.

## Introduction

Determining when research should be considered mature enough to support the establishment of a therapy is ethically complex, especially when considering deep brain stimulation (DBS) for psychiatric disorders, an invasive procedure that modulates brain function [1]. In this paper, we chart the evolution of DBS for treatment refractory obsessive-compulsive disorder (OCD) and present the consensus view of the Task Force on Neurosurgery for Psychiatric Disorders of the WSSFN on why this intervention can be considered an emerging and not an established therapy yet.

### History

The U.S. Food and Drug Administration (FDA) approved DBS for treatment-refractory OCD as a humanitarian device exemption (HDE H050003) in 2009. FDA-HDE approval does not require that applicants demonstrate efficacy, but applicants do need to show that the intervention does not pose unreasonable risks [2]. This FDA-HDE approval has been criticized, as the evidence for safety and efficacy of DBS in OCD at that time was limited [3].

Also in 2009, a Conformité Européenne (CE mark) was obtained by Medtronic Inc. and reimbursement was achieved in several, but not all, EU countries. However, CE mark at the time of approval only referred to the safety of a product for a particular use, i.e., DBS in the internal capsule and its vicinities using a particular DBS electrode from a particular provider. The CE mark states nothing about efficacy, patient selection criteria, etc.

In 2014, the Neurosurgery Committee for Psychiatric Disorders of the WSSFN published consensus guidelines for the use of stereotactic neurosurgical interventions to treat refractory psychiatric disorders [4]. The consensus statement noted that, “In this delicate field of neurosurgery for psychiatric disorders, it seems reasonable to state the following requirement before the surgical intervention can be stated as “approved therapy”. At least two blinded (if feasible) randomized controlled clinical trials from two different groups of researchers need to be published, both reporting an acceptable risk-benefit ratio, at least comparable with other existing therapies. The clinical trials should be on the same brain area for the same psychiatric indication.”

### Evidence

The Task Force on Neurosurgery for Psychiatric Disorders of the WSSFN, which is the successor organization to the Neurosurgery Committee for Psychiatric Disorders of the WSSFN, now recognizes that two such blinded randomized controlled trials have been published [5, 6]. Below is a brief summary of the results of these two studies, both using DBS of the ventral anterior capsule region for the treatment of refractory OCD.

Denys et al. reported on their patients with stimulation on (open label phase) for 8 months before randomization [5]. Nine out of 16 patients were responders, with a mean decrease of 46% in Yale-Brown Obsessive Compulsive Scale (Y-BOCS) score during the initial open-label phase. Following these 8 months, there was a 2-week cross-over phase, during which the Y-BOCS dropped 25% during active DBS versus sham, using a double-blind, sham-controlled design. While encouraging, this decrease in Y-BOCS score did not achieve a response rate generally considered to be a minimum of a 35% decrease in Y-BOCS score [7]. In summary, a significant albeit less than optimal effect was observed in the active stimulation phase (25% vs. the required 35%).

In a different study, Luyten et al. reported in 17 patients a response rate of 53% and a significant improvement (median: 37%) in Y-BOCS scores when comparing the blinded ON phase with the blinded OFF phase during a randomized cross-over trial [6]. During the open label phase, 67% of the patients were responders and the median decrease in Y-BOCS score was 58%.

Of note, these two studies targeted slightly different loci in the brain. Denys et al. targeted the nucleus accumbens [5]. However, as documented in subsequent publications, the active electrode contacts were located dorsal to this target, in the ventral anterior limb of the internal capsule [8]. Luyten et al. targeted the bed nucleus of the stria terminalis [6]. These structures are in very close proximity, and in both studies electrical contacts of leads of various designs were also located in the anterior limb of the internal capsule. Both studies reported acceptable safety and efficacy.

What level of evidence can we assert for the efficacy of this intervention in refractory OCD? Level I evidence implies, in practice, that “clinicians should follow the strong recommendation unless a clear and compelling rationale for an alternative approach is present” [9–11]. According to the Canadian Task Force on the Periodic Health Examination’s Levels of Evidence, one needs at least one randomized controlled trial (RCT) with proper randomization to obtain level I evidence [9]. Concerning the results of the Denys et al. study, in our opinion only the Luyten et al. study fulfills the Canadian Task Force requirement [5, 6]. Sackett et al. categorize outcomes as follows: level of evidence I requires RCTs with enough power and sample sizes large enough to achieve significance; level II signifies small RCTs with unclear results [12]. Taken together, we consider that Denys et al.’s study qualified for level II and Luyten et al.’s study qualified for level I, even though it included a small number of participants [5, 6]. We make this assertion because neuromodulation trials for OCD do not, and will never, command large numbers of participants and, for that reason, large RCTs (as, for example, in drug trials) are not feasible. While only a very small number of patients meet the inclusion and exclusion criteria for this kind of surgical intervention, a large effect was obtained by DBS in Luyten et al. paper consistent with level I evidence for efficacy of the therapy [6]. Moreover, the paper of Luyten et al. is considered as class I evidence for DBS in OCD according to Bari et al. [6, 13]. None of the authors of the paper of Luyten et al. co-authored the paper of Bari et al. [6, 13]. Recommending a GRADE level according to the GRADE Practice Recommendations would require an extensive effort and has not yet been achieved [14].

Furthermore, two non-randomized studies—the largest in this field—substantiate the results, obtained in the Luyten et al. paper [6, 15, 16]. Target was anterior internal capsular region, including bed nucleus of stria terminalis and/or nucleus accumbens. In a clinical cohort study of 70 patients, a 40% reduction of mean Y-BOCS scores was observed after 12 months of open label DBS, with 36 of the 70 patients being categorized as responders (52%), 12 patients as partial responders (17%), and 22 patients as non-responders (31%) [15]. Adverse events included transient symptoms of hypomania, agitation, impulsivity, and sleep disorders.

In a further open prospective international multi-center study, a mean Y-BOCS reduction of 42% at 12 months (*N* = 30) was obtained and the responder rate was 60% [16]. Most adverse events were mild or moderate, transient and related to programming/stimulation and tended to resolve by adjustment of stimulation parameters.

In summary, there are convincing beneficial effects of DBS on OCD symptoms in carefully selected patients who are refractory to expert psychological therapy (typically cognitive behavioral therapy) and rigorous pharmacological treatment.

This emerging consensus can be corroborated based on discussions at five recent scientific meetings (Congress of the WSSFN 2017, Berlin; Joint Meeting of the Neurosurgical Societies of Belgium and The Netherlands, Den Bosch, 2017; Congress of the Japanese Neurosurgical Society, October 2017; Neuromodulation, the rising trend in treating nervous system disorders, Samsun, Turkey, October 2017; Asia-Pacific Centre for Neuromodulation—DBS symposium, Brisbane, November 2017). Compared to ablative lesional surgery (bilateral anterior capsulotomy, bilateral cingulotomy, subcaudate tractotomy or limbic leucotomy), which has been performed and documented for decades, DBS offers the advantage of being partially reversible, at least in the short-term [17]. However, DBS for OCD remains an expensive treatment with a heavy clinical service work-load in terms of programming and life-long follow up, and it is not without risk [18, 19].

### Role of the psychiatrist

Notwithstanding the accumulating evidence of the effectiveness of DBS for OCD, and despite the need for alternative treatments for patients who do not respond to available psychological and pharmacological therapies, even when expertly delivered, only a few psychiatrists seem to take the initiative to consider neurosurgical options. The statement of Laitinen from 1977 remains valid: In a publication titled “Ethical aspects of psychiatric surgery” he wrote: “The basic problem of psychosurgery is psychiatric, therefore the initiative in considering surgical treatment must be taken by the psychiatrist. As soon as the psychiatrist is sure that conservative treatment by every available method cannot cure the patient, the psychiatrist should consult the neurosurgeon. Psychosurgery will remain experimental for years. Therefore, its use should be concentrated and restricted to psychosurgical research units having strong and intimate affiliation with scientists from many disciplines” [20]. That statement was made during the stereotactic lesional era, and remains valid today in the DBS era.

The Task Force on Neurosurgery for Psychiatric Disorders of the WSSFN reaches out to psychiatrists to improve communication between psychiatrists and neurosurgeons to carefully evaluate when it may be in the best interest of a patient to consider neurosurgery and to implement an ethical scientific program for surgical treatment of refractory OCD patients.

## Conclusion

*The Task Force on Neurosurgery for Psychiatric Disorders of the WSSFN accepts that electrical stimulation for otherwise treatment refractory obsessive-compulsive disorder using a multipolar electrode implanted in the region of the bed nucleus of the stria terminalis and/or anterior limb of the internal capsule represents an emerging therapy*.

This is based on the following data: one peer-reviewed scientific paper with level 1 evidence for electrical stimulation of this target region for treatment refractory patients with OCD [6], one peer-reviewed scientific paper with level 2 evidence [5], one large cohort study [15] and one open prospective international multi-center study [16].

Neurosurgery for psychiatric disorders is a sensitive topic in many parts of the world. For that reason, but also for clinical and ethical reasons, it is only logical that the requirements are set high for it to become “standard of care”. On the other hand, it would be unreasonable and imprudent to continue to view this approach as purely investigational once sufficient evidence of safety and efficacy are demonstrated to validate the intervention as a treatment.

This represents a step forward, but in order to meet the criteria outlined above, at least one additional, well-designed, blinded clinical trial will be necessary. Until then, surgery can be offered for refractory OCD based on the promising data obtained thus far, albeit with proper regulatory oversight. In the US, despite the FDA HDE approval statement from 2009, review by an institutional review board (IRB) remains necessary. It is our view that in whatever jurisdiction within which a surgeon practices, it is in the interest of patients and the integrity of the work that until DBS in OCD is fully vetted as an established therapy, surgeons consult their local IRB and/or research ethics committees when undertaking investigational procedures and as required by local jurisdictional laws and prevailing norms.

Surgery for OCD remains a treatment of last resort, and careful patient selection according to clear definitions of “treatment refractoriness” is recommended [21]. Clinicians and researchers are encouraged as much as possible to design and conduct randomized controlled trials to further evaluate this treatment as well as to investigate suitable brain targets for OCD and other psychiatric indications.

The Task Force affirms that it is incumbent upon psychiatrists to collaborate with surgeons and to drive this process, as have done the many neurologists worldwide who drove and are still driving the field of surgery for Parkinson’s, other movement disorders and epilepsy, both clinically and scientifically. The future of surgical treatment of psychiatric patients remains in the realm of the psychiatrist.
